# Coaching ward pharmacists in antimicrobial stewardship: A pilot study

**DOI:** 10.1016/j.rcsop.2022.100131

**Published:** 2022-03-29

**Authors:** Sharmila Khumra, Andrew A. Mahony, Kay Stewart, Phillip J. Bergen, Rohan A. Elliott

**Affiliations:** a Centre for Medicine Use and Safety, Faculty of Pharmacy and Pharmaceutical Sciences, Monash University, 381 Royal Parade, Parkville, Victoria 3052, Australia; b Department of Pharmacy, Austin Health, 145 Studley Road, Heidelberg, Victoria 3084, Australia; c Department of Infectious Diseases, Austin Health, 145 Studley Road, Heidelberg, Victoria 3084, Australia; d Department of Medicine, Building 181, University of Melbourne, Grattan St, Melbourne, Victoria 3010, Australia

**Keywords:** Antimicrobial stewardship, Education, Hospitals, Pharmacists, Coaching, Pilot projects, Feasibility studies

## Abstract

**Background:**

Ward pharmacists are well-positioned to enhance the activities of hospital antimicrobial stewardship (AMS) programs by reviewing the appropriateness of antimicrobials and making recommendations to prescribers. However, recent studies have identified gaps in ward pharmacists' AMS practice, knowledge, skills, and confidence which suggests education and training programs are needed.

**Objective(s):**

To describe, for the first time, an interactive educational activity – coaching in AMS – targeted at ward pharmacists and explore their perceptions of coaching as a mode of delivering education to improve AMS knowledge, skills, confidence, and practice. A secondary objective was to describe the type, frequency, and acceptance of AMS recommendations made by coached pharmacists.

**Methods:**

This was a descriptive pilot study with a qualitative evaluation of pharmacists' perceptions and experiences of coaching. AMS coaching was delivered over 2 months in 2019 to pharmacists providing clinical pharmacy services to general medical and surgical wards. A focus group was conducted one month after the coaching period to elicit pharmacists' perceptions of coaching as a mode of delivering AMS education and how it impacted their AMS knowledge, skills, confidence, and practice. AMS recommendations made by coached pharmacists were prospectively recorded, and the prescriber acceptance rate was determined.

**Results:**

Ward pharmacists reported positive experiences with AMS coaching and believed it helped them identify a range of recommendations to improve antimicrobial prescribing and increased their confidence to communicate recommendations to prescribers. Workload issues were identified as the main barrier to implementation. Suggestions were provided to improve coaching implementation feasibility. During coaching, 162 AMS recommendations were identified for a range of antimicrobials, and 69% (113/162) were accepted and implemented.

**Conclusions:**

Ward pharmacists believed coaching improved their AMS knowledge, skills, confidence, and practice, including their confidence to discuss recommendations with prescribers. These results can assist with the design and evaluation of future hospital-based AMS educational initiatives.

## Highlights

• Ward pharmacists are well-placed to contribute to antimicrobial stewardship (AMS).
• Coaching may address gaps in pharmacists' AMS knowledge, skills and confidence.
• Ward pharmacists' perceptions of AMS coaching were generally positive.
• Coaching helped pharmacists enact AMS recommendations to improve prescribing.
• Further research into effectiveness of coaching on patient outcomes is needed.

## 1 Introduction

Antimicrobial stewardship (AMS) programs are increasingly utilised in hospitals to address rising rates of antimicrobial resistance and reduce the risk of adverse effects associated with antimicrobial use (e.g., renal toxicity, antibiotic allergy, and secondary infection with *Clostridioides difficile*).^1^^,^^2^ AMS programs promote safe and appropriate antimicrobial prescribing and have been shown to improve patient safety and clinical and economic outcomes.^3^^,^^4^^,^^5^ Typically, an AMS program involves a centralised approach whereby a multidisciplinary team, which generally includes an infectious disease (ID) physician and a pharmacist knowledgeable in ID and AMS (ID/AMS pharmacist), is dedicated to coordinating AMS activities at a hospital-wide level.^6^ Prospective audit and feedback (PAF) is a core AMS activity involving the AMS team reviewing antimicrobial therapy after prescription, with real-time discussion of recommendations with the prescriber to optimise prescribing.^1^ Recommendations can include discontinuing therapy, switching from intravenous to oral antimicrobials (IV-to-oral switch), de-escalation such as changing to a more narrow spectrum of antimicrobial based on the results of microbiology tests, and dose optimisation.^2^ Due to limited human resources and time, AMS teams are usually unable to review the prescribing of all antimicrobials in all clinical areas, and therefore they usually target certain antimicrobials (e.g., broad-spectrum agents because of their potential to select for resistant organisms), specific infections (e.g., *Staphylococcus aureus* bacteraemia), and clinical units with high use of antimicrobials (e.g., intensive care or haematology units).^1^^,^^2^

To broaden AMS coverage, education and training of frontline clinicians (doctors, pharmacists, and nurses) involved in antimicrobial prescribing, dispensing, and administration is recommended.^7^^,^^8^^,^^9^ Ward pharmacists are well-positioned to extend the reach of PAF as their role entails review of prescribed medicines and provision of advice to doctors regarding safety and appropriateness.^10^ However, AMS is not a mandatory component of the undergraduate pharmacy curriculum in Australia,^11^ and a recently published Australian survey of 439 hospital pharmacists identified gaps in their AMS practice, confidence and knowledge.^12^ Fifty percent or fewer respondents were confident in identifying AMS interventions related to dose optimisation based on infection-specific factors, bug-drug mismatch (i.e., inappropriate microbe and antimicrobial combination), and inappropriate lack of spectra of antimicrobial activity. Key knowledge gaps were noted in antimicrobials' anaerobic spectrum and beta-lactam allergy assessment. Another recent survey of 553 ward pharmacists in Malaysia reported a moderate level (median score of 3 out of 5) of self-perceived competence in AMS, including knowledge on antimicrobial use, therapeutic management of infections, and interpretation of laboratory tests and/or disease markers.^13^ More than half of the respondents cited a lack of training as a major barrier to participating in AMS.

Most hospital-based AMS educational initiatives reported in the literature are directed towards improving doctors' prescribing.^14^ These initiatives typically target specific antimicrobials,^15^ or infections,^16^ or aim to improve the practice of specific AMS interventions such as IV-to-oral switch or discontinuing therapy when the recommended duration has been reached.^16^^,^^17^ While PAF led by ward pharmacists to reinforce prescriber education is occasionally included in these studies, the training provided to these pharmacists is seldom described, and the scope of the intervention is limited to the target antimicrobials or interventions. Only two published studies have evaluated the effectiveness of educational interventions targeted at hospital pharmacists to improve their AMS knowledge and skills to perform PAF on a wide range of antimicrobials and infections.^18^^,^^19^ Importantly, these studies provided very limited evaluation of learners' perceptions of the benefit of the education they had received using only 5-point Likert scale survey questions^18^ or yes/no questions.^19^

AMS educational strategies that incorporate interactive or dynamic techniques such as audit and feedback or one-on-one targeted sessions are considered more effective than passive techniques (didactic programs, lectures, written material, etc.) to change behavior and improve processes.^9^^,^^10^ In the Australian pharmacist survey mentioned above, interactive modes of education delivery such as a rotation in ID/AMS under supervision, one-on-one mentoring/coaching, and interactive small groups were perceived by a higher proportion of respondents to be useful or very useful than more didactic options such as lectures and e-learning modules.^12^ Similarly, in the Malaysian study, a higher proportion of survey respondents preferred to learn about AMS via hands-on, experiential, or mentoring activities.^13^ This pilot study aimed to describe for the first time the use of an interactive educational coaching approach to AMS targeted at ward pharmacists. The pharmacists' perceptions of coaching as a mode of delivering education to improve their AMS knowledge, skills, confidence, and practice were explored, as well as the feasibility of implementing such an approach. A secondary aim was to describe the type, frequency, and acceptance of AMS recommendations made by pharmacists who received coaching.

## 2 Methods

### 2.1 Design

This was a descriptive pilot study, with qualitative evaluation of pharmacists' experiences and perceptions. It was approved by the Human Research Ethics Committees of the participating hospital and university (approval numbers LNR 55547/2019 and 22,331, respectively).

### 2.2 Setting and participants

The study was conducted at a tertiary care public hospital in Melbourne, Australia, which has over 560 acute beds. The hospital has a long-established, inter-disciplinary AMS program that utilises key AMS strategies, including a hospital-wide preapproval system for target antimicrobials and PAF by the AMS team focussed on certain clinical units (intensive care, spinal, diabetic foot and plastic surgery) and specific antimicrobials (e.g., systemic antifungals). At the time of the study, there were 2.4 full-time equivalent ID/AMS pharmacists. All clinicians (doctors, pharmacists, and nurses) in the hospital have electronic access, via the hospital's intranet, to national antimicrobial prescribing guidelines (Therapeutic Guidelines: Antibiotic),^20^ and local antimicrobial prescribing guidelines for selected antimicrobials/infections, and IV-to-oral switch.

Clinical pharmacy services on the four study wards were delivered between 08:30 and 17:30, Monday to Friday, by one ward pharmacist for each 32-bed ward. The clinical pharmacy model is ward-aligned, and pharmacists do not routinely attend ward rounds. Weekend clinical pharmacy services were limited and excluded from this study. As part of their usual weekday activities, ward pharmacists were expected to review prescribing of all medications, including antimicrobials, to ensure safety and appropriateness. Pharmacists were expected to ensure that the prescriber obtained a valid preapproval before supplying targeted (restricted) antimicrobials. At the commencement of employment at the hospital, all ward pharmacists at the study hospital receive orientation on its AMS program, including their responsibilities associated with the antimicrobial approvals system. They are also provided with two pocket-size cards which outlined 1) recommended empirical antibiotics and dose regimens for common medical and surgical infections, and 2) clinical criteria for IV-to-oral switch and recommended equivalent oral antimicrobial regimens; both cards were introduced several years prior to the present study. Pharmacists may also receive occasional brief ID/AMS in-service education on an ad hoc basis via the Pharmacy Department's Continuing Education program. The pharmacists who participated in this study were given no specific in-house training in AMS in the lead-up to this study.

Ward pharmacists who provided weekday clinical pharmacy services to general medicine and general surgery patients on four wards and pharmacists rostered to cover these wards if the usual pharmacist was on leave were invited to participate in this study by one of the researchers. These wards were chosen because there was no PAF intervention provided by the AMS team (other than for systemic antifungals).

### 2.3 Description of AMS coaching and its implementation

Coaching occurred over 8 weeks (October 28 to December 20, 2019) and involved one-on-one sessions with ward pharmacists working on the four study wards. Sessions were planned to be delivered 2 to 3 times per week for each ward pharmacist, Monday to Friday, at times suitable to the ward pharmacist, with the aim of taking no longer than 15 min per session (i.e., it was anticipated each pharmacist would receive ~4–6 h of coaching across the 8-week period). All coaching was delivered by one pharmacist (the AMS coach) with more than 10 years of practical experience in ID/AMS and prior experience in research and tertiary clinical pharmacy education. Coaching sessions were conducted on the wards where the pharmacists worked.

Coaching sessions involved discussions about patients admitted to general medicine or general surgery units on the four study wards who were receiving systemic antimicrobial(s). During coaching, systemic antimicrobials prescribed for an extended duration (≥28 days), medical prophylaxis, or non-infective indications were not discussed. Target antimicrobials with an ‘interim’ approval (24-h approval for non-standard indications, which were reviewed daily by the ID/AMS pharmacist) and antifungals targeted for weekly PAF by the AMS team were also not discussed.

Patients were identified from an electronic antimicrobial order report generated by the AMS coach for each ward on the day of coaching. The discussions focused on AMS principles and opportunities for intervention to improve prescribing, i.e., ensuring the right antimicrobial, at the right dose regimen, by the right route, and for the right duration for the right patient. All aspects of AMS were covered utilising the 4 moments of antibiotic decision-making tool adapted for pharmacists.^21^ During coaching, ward pharmacists were asked to: determine what infective syndrome the patient had by reviewing their signs and symptoms and clinical notes; determine whether the prescribed empirical therapy followed national or local guidelines; interpret reports of relevant laboratory tests including microbiology culture and sensitivity tests and radiology tests, and apply the findings to the patient (for example, reviewing the radiologist comments for an abdominal computerised tomography [CT] to determine if the patient has a perforation or abscess requiring a longer duration of antimicrobial therapy); identify potential recommendations to improve antimicrobial prescribing; and review the duration of therapy to ensure compliance with national or local guidelines. Ward pharmacists were encouraged to refer to the pocket-size cards and local antimicrobial prescribing guidelines and shown how to apply information in these resources to assess the appropriateness of antimicrobial prescribing. After each coaching session, the ward pharmacist contacted the treating medical or surgical team to propose AMS recommendations that had been discussed.

During the sessions, the AMS coach aimed to uncover any gaps in AMS knowledge, skills, and performance and then target these areas for discussion in subsequent coaching sessions. After a period of time, dependent on the ward pharmacists' level of knowledge and skill, the AMS coach did less talking and encouraged ward pharmacists to lead a discussion of the patient's infection management and appropriateness of antimicrobial prescribing. The AMS coach guided discussions when necessary to enable the ward pharmacist to formulate AMS recommendations independently.

### 2.4 Data collection

#### 2.4.1 Qualitative data - Ward pharmacists' perceptions of AMS coaching

One month after the end of the coaching period, ward pharmacists who had participated in coaching were invited to attend a focus group to elicit their perceptions and experiences with coaching to deliver AMS education. Participation in the focus group was voluntary, and no incentives were offered. A focus group session was chosen as they are an efficient way to collect data, with the benefit of allowing participants to share their ideas. An interview guide [Supplementary material] was developed to aid the discussion and guide the conversations around the topics of interest to the researchers. These were: ward pharmacists' experiences with coaching, particularly the impact they felt it had on their AMS knowledge, skills, confidence, practice, and how coaching compared with other modes of AMS education delivery. Additional questions were asked about their opinions on the feasibility of receiving coaching in the workplace. The session was facilitated by a researcher experienced in qualitative research (KS) who was not the pharmacist who delivered coaching and not a hospital employee. An assistant took notes, and the session was audio-recorded using iPads. The focus group lasted 45 min.

#### 2.4.2 Quantitative data - Ward pharmacists' AMS recommendations

The AMS coach recorded the number of coaching sessions per ward pharmacist and the duration (minutes) of each session. The AMS coach prospectively recorded all AMS recommendations identified by pharmacists during the coaching session. The AMS coach contacted the ward pharmacist the day after the coaching session to determine which AMS recommendations were discussed with the treating team. The AMS coach then reviewed the electronic medication record (EMR) to check if the recommendations were implemented within 24 h. AMS recommendations discussed during coaching but not discussed by the ward pharmacist with the treating team were noted, with an accompanying reason for not having been discussed. AMS recommendations discussed with the treating team were categorised according to the ‘5 moments of antimicrobial prescribing’ (escalation, de-escalation, discontinuation, switch and optimise therapy).^22^

### 2.5 Data analysis

#### 2.5.1 Qualitative data - Ward pharmacists' perceptions of AMS coaching

The audio-recording was transcribed verbatim by a professional transcribing company and checked for accuracy against the recording by two researchers before they undertook independent content analysis. Content analysis was based on six predetermined topics that formed the interview guide, namely impact of coaching on AMS 1) knowledge, 2) skills, 3) confidence, 4) practice, 5) how coaching compared with other modes of AMS education delivery, and 6) implementation feasibility. The first five topics were selected because they reflect the potential outcomes or benefits of learning/education based on the Kirkpatrick evaluation model.^23^ Implementation feasibility was also included to explore the practicality of AMS coaching given this was a new mode of AMS education delivery. The researchers also looked for additional unexpected topics or themes.

#### 2.5.2 Quantitative data - Ward pharmacists' AMS recommendations

The number of coaching sessions per ward pharmacist and ward, and time taken for each session, were summarized using median and interquartile range (IQR). Descriptive statistics regarding AMS recommendations, acceptance, and antimicrobials were summarized using frequency and percentages. Statistical analyses were performed using Microsoft Excel (Microsoft Corporation, 2018).

## 3 Results

### 3.1 AMS coaching sessions

Over the 8-week study period, eight ward pharmacists participated in coaching. All were female with two to six years of experience post-registration. Two had completed an elective unit in ID/AMS as part of a postgraduate certificate in pharmacy practice approximately 5 years prior to the study. A total of 70 coaching sessions were delivered (median two per ward per week, IQR: 2–3). There were 38 sessions delivered to four ward pharmacists who serviced the two general medical wards over the study period and 32 sessions to four ward pharmacists who serviced the two general surgical wards. The median number of coaching sessions per ward pharmacist was seven (IQR: 5–12). The overall median coaching time was 20 min (IQR: 15–30), which was similar across general medical and surgical wards (median time of 20 min (IQR: 15 to 30) for general medical wards and 20 min (IQR: 20 to 30) for general surgical wards). The AMS coach spent approximately 15 min per session generating an antimicrobial order report for the ward, contacting the ward pharmacist, and walking to and from the ward.

### 3.2 Qualitative analysis: Ward pharmacists' perceptions of AMS coaching

All eight ward pharmacists who participated in the coaching sessions also participated in the focus group session. Content analysis retained the six predetermined topics and did not identify any additional topics. In some cases, information discussed under a particular topic was transferred to a more appropriate topic.

#### 3.2.1 Perceptions of coaching impacting on AMS knowledge

Ward pharmacists were initially asked to comment on how coaching impacted their knowledge of appropriate antimicrobial use and provide examples. A majority of the participants provided comments that suggested coaching helped improve their knowledge. Specific examples where knowledge had improved included the appropriate duration of antimicrobial therapy post-surgery and test results requiring review to assess ongoing appropriateness of antimicrobial prescribing (i.e., microbiology, blood, and radiology tests):

“…[coaching discussed] *what's happened with your patient, like if they've had surgery they've now got source control, and so then it changes how you consider whether or not they need* [ongoing] *antibiotics. That's quite useful.” [pharmacist 2, 6 years post-registration, surgical].*

Pharmacists commented that coaching improved their knowledge on local rates of antimicrobial resistance and local antimicrobial prescribing guidelines that helped explain deviations from the national guidelines:

*“The example I can think of is about, more about certain resistance, like ‘Oh, this is shown to be more resistant to this, so this is what we use, this agent, even though the reference* [national] *guideline might not say that.’ So some extra local knowledge about things that you wouldn't necessarily find in* [national] *guidelines…” [pharmacist 4, 6 years post-registration, medical].*

“…*I was shown that there are a lot of local guidelines that I didn't know existed, so that was very helpful. And a lot of the guidelines also have treatment durations in there which I didn't know, so that helped a lot as well.” [pharmacist 8, 6 years post-registration, surgical].*

#### 3.2.2 Perceptions of coaching impacting AMS skills

In general, ward pharmacists felt coaching helped improve their skills in interpreting microbiology and radiology test results, as well as changes in laboratory markers and signs and symptoms, and their ability to use this information to review the appropriateness of antimicrobial prescribing:

*“I guess it's looking at some of the radiology scans as well. So that's something that we didn't really do before, but that was something that we were coached to look at – particular scans and things, as well as different* [clinical] *observations …” [pharmacist 2, 6 years post-registration, surgical].*

A junior pharmacist commented that interpreting microbiology and radiology test results were not taught in undergraduate education:

*“…like you know how to really read the microscopy, read the radiology reports…it wasn't really taught in detail until we had that coaching.” [pharmacist 5, 2 years post-registration, medical].*

#### 3.2.3 Perceptions of coaching impacting AMS confidence

Pharmacists commented that coaching also helped improve their confidence to discuss AMS recommendations with prescribers:

*“Feeling more confident in duration, because we're able to have the knowledge about how long it should actually be.” [pharmacist 1, 2 years post-registration, medical].*

*“I think maybe it's* [coaching] *given us a bit more confidence sometimes to go up to the doctors and say ‘Oh, you know based on these facts…’, and some more objective results like looking at CRP, the fact they've been afebrile for this many days, kind of gives you a bit more confidence to make clinical recommendations." [pharmacist 2, 6 years post-registration, surgical]*

Pharmacists who only received coaching for a short period of time (1–3 weeks, because they were covering the ward during a period of leave) believed their confidence to communicate AMS recommendations to prescribers might have improved if they were coached for a longer period. Also, pharmacists commented that while coaching improved their confidence to review antimicrobial prescribing for the types of patients under their care at the time of the coaching, they were unsure if they would feel as confident reviewing prescribing for different patient groups when rotated to another clinical area:

*“I got quite good at knowing the general medicine sort of antibiotics and durations and that sort of thing. But not necessarily more surgical type of patients.” [pharmacist 7, 2 years post-registration, medical].*

#### 3.2.4 Perceptions of coaching impacting future AMS practice

Pharmacists discussed how their practice changed post-coaching. For example, some commented that they were reviewing radiology test results (which they had not routinely performed previously) to determine the appropriateness of prescribing and having greater awareness of certain AMS recommendations:

*“I look at radiology a bit more in taking into account whether that affects duration of therapies; for example, if it's just a simple UTI versus pyelonephritis which can be picked up on the CT.” [pharmacist 5, 2 years post-registration, medical].*

*“Yeah, I think it's* [coaching] *made us more conscious of things, like I'm more conscious about telling or asking prescribers to switch from IV to oral or stopping antibiotics on discharge.” [pharmacist 6, 3 years post-registration, surgical].*

#### 3.2.5 Perceptions of coaching as a mode of education delivery

Pharmacists were asked how coaching compared to other modes of education delivery such as lectures, guidelines, and pocket cards, for learning about antimicrobial use and AMS. Pharmacists commented that a positive aspect of coaching was the ability to immediately apply AMS principles to practice by discussing antimicrobial prescribing for patients currently under their care:

*“It's good because it's a targeted approach…about the patient that you're looking after, the patient in front of you, so it puts more of the principles that you would otherwise just learn into practice a little bit more.” [pharmacist 5, 2 years post-registration, medical].*

*“…coaching helps talk you through than more just reading something and trying to figure out how it applies to your patient.” [pharmacist 4, 6 years post-registration, surgical].*

Negative aspects of coaching were also raised. In general, pharmacists felt that coaching was difficult to fit into their day, particularly for wards with high patient turnover, such as surgical wards:

*“It's* [coaching] *a good approach …. But it's just not sustainable, because of the workload and how the wards are run, because sometimes you can't do it. You've got discharges, you've got admissions. So sometimes it was actually hard to sit down and do it.” [pharmacist 3, 5 years post-registration, surgical].*

#### 3.2.6 Suggestions to improve feasibility of coaching

In general, the participants felt that the number of coaching sessions (2 to 3 times per week) and the time taken for coaching (median 20 min) were challenging to accommodate. To improve time efficiency, it was suggested to target certain antimicrobials (e.g., intravenous or antimicrobials requiring preapproval) or certain patients, rather than reviewing all antimicrobial orders. Both experienced and less-experienced pharmacists suggested targeting interns or newly registered pharmacists to maximise improvements in AMS knowledge, skills, and confidence:

*“…maybe* [coaching] *could be incorporated into teaching of intern pharmacists for example, so they have that knowledge from the start, and they can therefore apply the principles from the get-go…” [pharmacist 5, 2 years post-registration, medical].*

*“…junior pharmacists would be the best target, they would filter those* [AMS] *concepts, so you start being aware of them as an intern” [pharmacist 1, 6 years post-registration, medical].*

Less frequent one-to-one coaching and group coaching were also proposed. It was suggested that group coaching would enable the review of different types of patients, for example, both medical and surgical patients, to help broaden knowledge of different infections and AMS recommendations. Most pharmacists agreed that certain aspects of infection management, such as interpreting microbiology or therapeutic drug monitoring test results, could be discussed in a group, whereas more patient-specific factors could be discussed with individual pharmacists on the ward. Some pharmacists, nevertheless, expressed a preference to receive all AMS coaching one-to-one in their work area:

*“I like that the coaching was done on the ward as well, so if we were reviewing a patient* [with the coach]*, weren't sure about something, we can grab the notes, have a read of what's been happening over the last couple of days. So, it's good to have it* [coaching] *in that environment where you normally work.” [pharmacist 2, 6 years post-registration, surgical]*.

*“And the doctors are nearby* [during coaching] *so you can turn around and ask the doctors walking by, ‘Oh what's the plan, what's happening?’ So, it's good, it's quite hands on.”* [*pharmacist 1, 2 years post-registration, medical].*

### 3.3 Quantitative analysis: Ward pharmacist AMS recommendations

A total of 558 systemic antimicrobial orders for 385 patients were reviewed across all coaching sessions. A further 77 antimicrobial orders were not reviewed due to a lack of time (*n* = 34), the patient being discharged (*n* = 28), or the order being ceased (*n* = 16) prior to the coaching session. For 35.1% (196/558) of the reviewed antimicrobial orders (involving 156 patients), 214 potential AMS recommendations were identified. Of these, 52 were excluded from analysis because the ward pharmacist did not discuss them with the clinical team due to: 1) competing clinical priorities/lack of time (*n* = 18); 2) the recommended change to therapy was self-initiated by the treating team (*n* = 23); or 3) the change was escalated by the ID/AMS pharmacist (coach) to an ID or microbiology physician for review and intervention (e.g., release of further antimicrobial susceptibility test results or preapproval system rules not being followed) (*n* = 11).

**Fig. 1:**
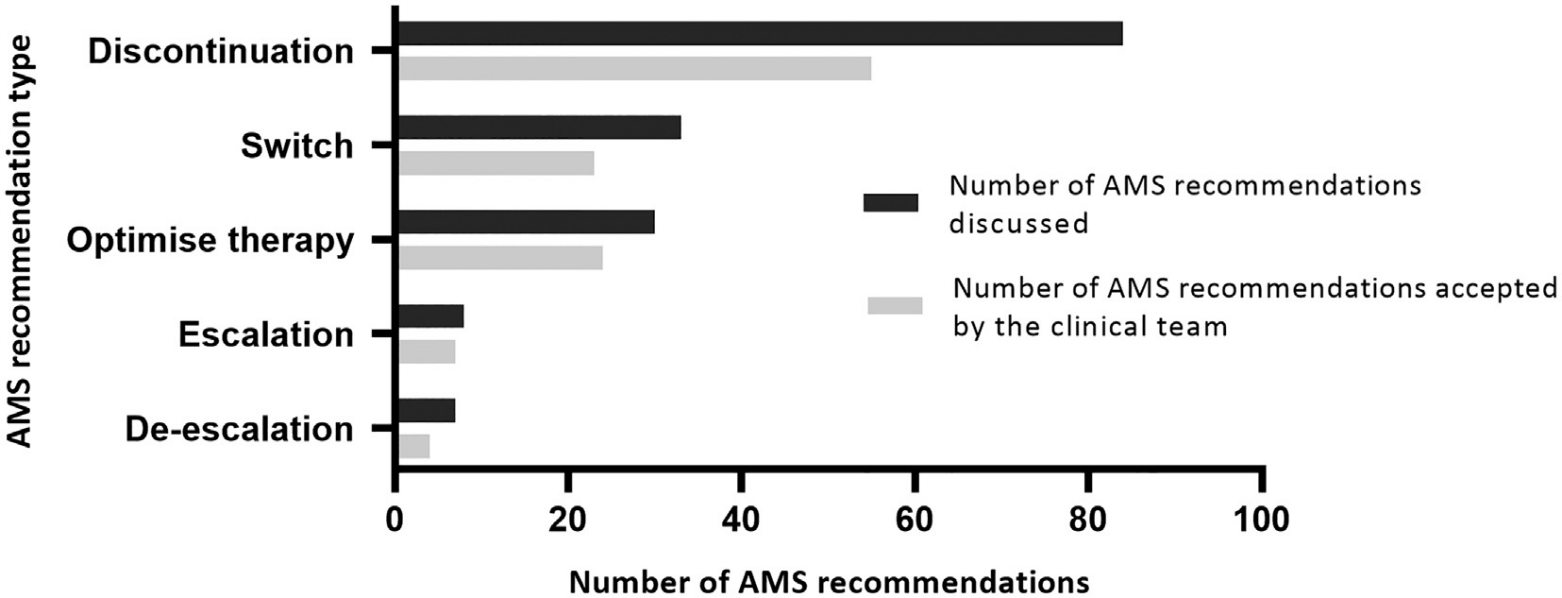
Type, frequency, and acceptance of antimicrobial recommendations made by ward pharmacists following antimicrobial stewardship (AMS) coaching sessions. Recommendations included in each of these categories were: Discontinuation: cease an antimicrobial due to unlikely infection, recommended duration of therapy reached, unnecessary spectrum of antimicrobial activity, entering a cease date in the electronic medication record (EMR); Switch: switch from intravenous to oral antimicrobials; Optimise therapy: modify the dose regimen based on patient or infection related factors, management of drug-drug interactions, additional microbiology tests or therapeutic drug monitoring required; Escalation: broaden the spectrum of activity based on guidelines or microbiology test results; De-escalation: narrow the spectrum of activity based on guidelines or microbiology test results.

**Fig. 2:**
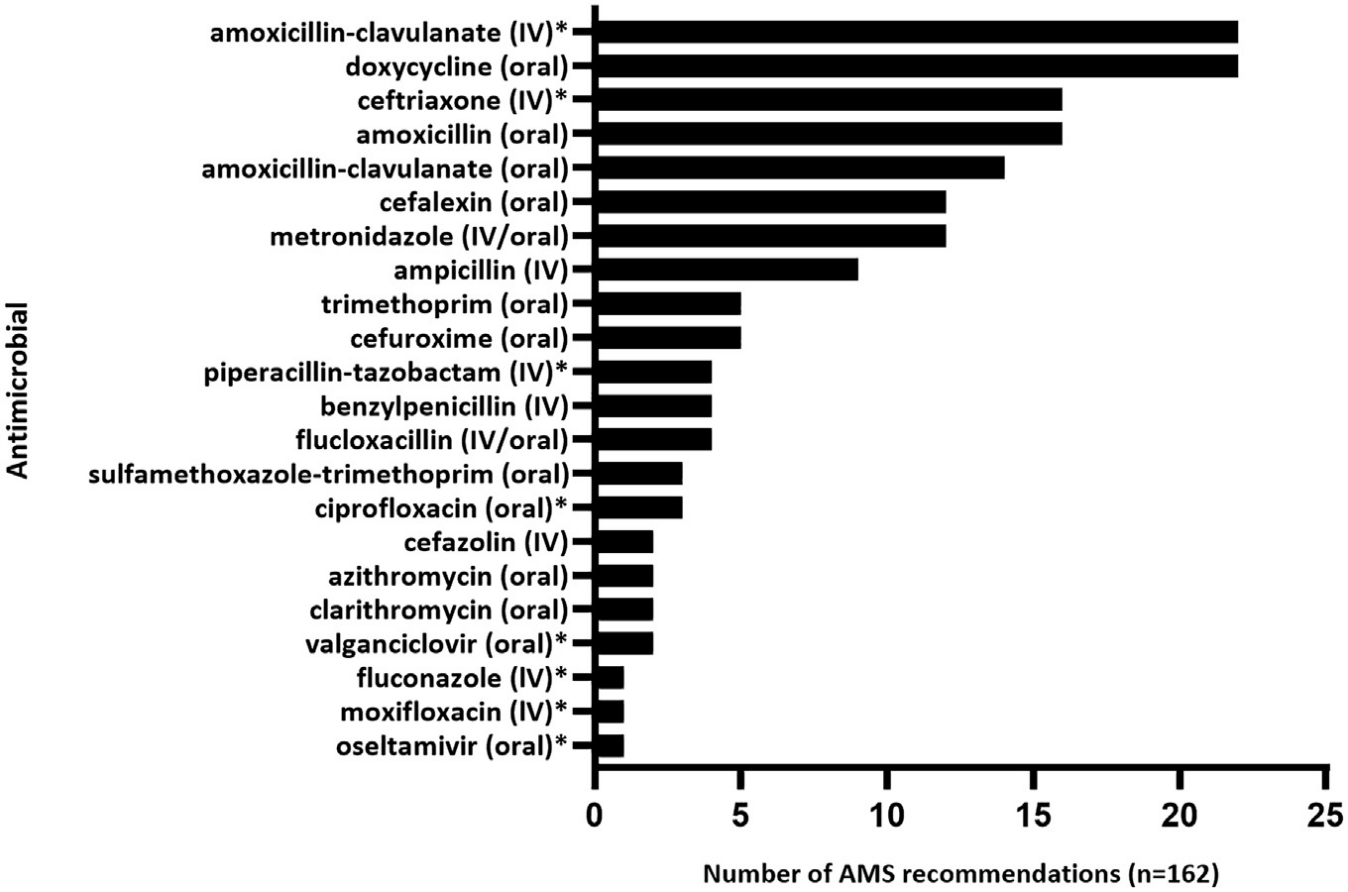
Number of AMS recommendations by antimicrobial. * Requires preapproval from an infectious diseases physician or electronic antimicrobial approvals system.

The type, frequency, and acceptance of the 162 AMS recommendations made by ward pharmacists following coaching sessions are shown in Fig. 1. The number of AMS recommendations by antimicrobial is shown in Fig. 2. Eight out of the top 10 antimicrobials by number of AMS recommendations (comprising 95 of the 162 [58.6%] recommendations) were agents not targeted by the hospital's AMS preapproval system. Within 24 h of ward pharmacist contact with the treating unit, the overall acceptance rate was 69.8% (113/162).

## 4 Discussion

This study described for the first time the use of an AMS coaching program targeting ward pharmacists on general medical and general surgical wards. Focus group data revealed that most ward pharmacists felt coaching improved their AMS knowledge, particularly which test results to review to assess the appropriateness of antimicrobial prescribing. Pharmacists stated that coaching improved their ability to interpret microbiology and radiology reports and use this information to determine the optimal choice of antimicrobial agent or duration, something they had not done well before coaching. Some ward pharmacists reported a heightened awareness of certain AMS interventions (IV-to-oral switch and discontinuing therapy), while all pharmacists felt coaching had made them more confident to discuss clinical information such as results of blood, microbiology, and radiology tests with the prescriber to support their AMS recommendation.

Regarding coaching as a method of AMS training, most participants felt that one-to-one coaching was more effective than less interactive or passive modes of education such as lectures or dissemination of guidelines, primarily because of the opportunity it afforded to apply AMS principles immediately to patients currently under their care. While setting aside time for AMS education was generally viewed positively, many found the time necessary to participate in the coaching sessions challenging to accommodate because of the need to prioritise frequent admissions and discharges. The median time for coaching was 20 min which was considered too long when sessions were held 2–3 times a week. The sessions were longer than anticipated when the study was planned (up to 15 min). This can be explained by the high frequency of antimicrobial prescribing in these units (particularly surgical units) and that some pharmacists wanted to discuss patients admitted to non-study units (‘borders’) or other aspects of infection management. Reviewing a specific number of patients, such as 3 to 5 patients, may be a way to shorten the time taken for coaching. Suggestions from participants to improve the feasibility of coaching included less frequent coaching sessions, restricting sessions to reviewing only certain antimicrobials or patients, and coaching in a group. However, on this latter point, some ward pharmacists stated a preference for one-on-one coaching. Most ward pharmacists felt intern (pre-registration) or newly graduated pharmacists would benefit more from coaching to better integrate AMS in their practice at the beginning of their career. Consistent with this, a recent national survey of Australian hospital pharmacists determined that those with two years or less of registration lacked the confidence to identify AMS interventions compared to more experienced hospital pharmacists.^12^ However, in our study, pharmacists with more than two years of experience also reported benefits from coaching.

In this study, with the assistance of an AMS coach, ward pharmacists were able to identify AMS recommendations for 35% of antimicrobial orders reviewed during coaching. The proportion of antimicrobial orders requiring AMS intervention reported here is lower than a Canadian study. The authors reported that 75% (80/106) of the antimicrobials reviewed by ward pharmacists were associated with at least one intervention.^24^ However that study was conducted at a hospital with no AMS program, focussed on a limited range of antimicrobials, and the most common interventions related to pharmacokinetic monitoring, a service led by the hospital pharmacy department. The lower rate in this study is not unexpected, given the study was conducted at a hospital with a well-established AMS program that included preapproval for selected antimicrobials and included most antimicrobial orders. The study hospital had good baseline antimicrobial prescribing appropriateness (overall rate of appropriateness 75% according to yearly antimicrobial point prevalence studies [unpublished data]) and good IV-to-oral switch practice.^25^

The AMS recommendations made by the participating pharmacists in the present study involved a wide range of systemic antimicrobials, most of which were antimicrobials that are not the usual focus of AMS programs. For example, recommendations frequently involved narrow-spectrum antimicrobials (IV or oral) such as ampicillin and doxycycline and oral broad-spectrum antimicrobials such as amoxicillin-clavulanate. Importantly, a number of these agents are included in the ‘curb’ category of Australia's Priority Antibacterial List for Antimicrobial Resistance Containment (i.e., antimicrobials commonly used as first-line agents for bacterial infections despite high antimicrobial resistance potential, e.g., oral amoxicillin-clavulanate)^26^; optimising their clinical use may have a significant impact on reducing the emergence of antimicrobial resistance. Results from the 2019 Australian hospital National Antimicrobial Prescribing Survey showed that just over half of all amoxicillin-clavulanate prescriptions (*n* = 1745) were non-compliant with guidelines, and more than a third of these prescriptions were deemed inappropriate overall.^26^ Cefalexin, another ‘curb’ antimicrobial associated with potentially missed AMS opportunities by hospital pharmacists regarding unnecessary or prolonged treatment,^12^ was another common target of the AMS recommendations in this study. Thus, with AMS coaching, ward pharmacists improved the appropriateness of commonly prescribed ‘curb’ antimicrobials.

Discontinuing antimicrobial therapy because of incorrect duration is a frequently missed opportunity to improve prescribing.^27^^,^^28^ In the 2019 Australian hospital National Antimicrobial Prescribing Survey, incorrect duration was one of the most common reasons for a prescription being assessed as inappropriate (24.1%, *n* = 5770 prescriptions).^27^ Similarly, in the current study, discontinuing antimicrobial therapy was the most common AMS recommendation made by ward pharmacists following coaching. Typical examples included discontinuing therapy because of no evidence of infection or the recommended duration for an infection had been reached. Importantly, the participating pharmacists indicated that coaching improved their ability to determine the appropriate therapy duration for a range of antimicrobials typically encountered on their ward. This key AMS intervention minimises unnecessary exposure to antimicrobials and reduces the risk of toxicity and the emergence of antimicrobial resistance.

There are some limitations to this study. Given it was undertaken in a single hospital with a well-established AMS program, findings may not be generalisable to other hospitals with less established or comprehensive AMS programs. However, in such hospitals, AMS coaching may yield greater benefits than reported here. Due to roster changes and periods of staff leave, most participating pharmacists received coaching for a shorter period than planned (hence the median number of sessions was only seven per pharmacist over the 8-week study period). This may have reduced the benefits derived by individual pharmacists. Pharmacist perceptions were obtained one month after coaching, and the longer-term impact on their practice was not examined. Some ward pharmacists may have felt uncomfortable providing negative feedback on the program in a group format. Only one AMS coach, who had extensive experience in ID/AMS and was known to the participants, may have positively influenced the study's findings. In further studies examining the wider implementation of AMS coaching, multiple coaches may be required, and training of coaches may be needed to ensure consistency. Finally, as this was a descriptive pilot study, no control group/ward was not exposed to AMS coaching or data collected on ward pharmacists' AMS recommendations prior to coaching. Therefore, it is uncertain what proportion of the recommendations would have been made without coaching. However, only recommendations directly related to the coaching sessions and not recommendations initiated by ward pharmacists prior to or between sessions or for other patients were reported. Importantly, the feedback from coached pharmacists highlighted that they would not have been able to make many of the recommendations without the extra knowledge and confidence attained through coaching.

In summary, this pilot study contributes to the existing literature on educational programs in AMS by describing an interactive mode of education delivery (coaching) targeted at ward pharmacists to improve their AMS knowledge, confidence, skills, and practice. Ward pharmacists had a positive experience with AMS coaching and believed it helped them identify a range of recommendations to improve antimicrobial prescribing and increased their confidence to communicate those recommendations to the prescriber. Incorporating regular coaching sessions into an already busy workload was seen as the main barrier to implementation. Further research is required to determine the effect of coaching wards pharmacists on antimicrobial use, antimicrobial prescribing appropriateness, and patient outcomes. Studies evaluating the resources required to implement coaching, its cost-effectiveness, and whether it has a sustained impact on ward pharmacists' ability to better integrate AMS principles in their clinical practice compared with other less intensive modes of education delivery are also required.

## Funding

This research did not receive any specific grant from funding agencies in the public, commercial, or not-for-profit sectors.

## CRediT authorship contribution statement

**Sharmila Khumra:** Conceptualization, Methodology, Investigation, Data curation, Writing – original draft. **Andrew A. Mahony:** Supervision, Writing – review & editing. **Kay Stewart:** Investigation, Writing – review & editing. **Phillip J. Bergen:** Supervision, Writing – review & editing. **Rohan A. Elliott:** Supervision, Methodology, Writing – review & editing.

## Declaration of Competing Interest

None.

## Appendix A Supplementary data

AMS coaching focus group interview guide Image 1
