# Supplementary material for: Coaching ward pharmacists in antimicrobial stewardship: A pilot study
Source: Explor Res Clin Soc Pharm. 2022 Mar 29;5:100131. doi: 10.1016/j.rcsop.2022.100131 (PMC9031758; doi:10.1016/j.rcsop.2022.100131)
Supplement: Supplementary file 1 — AMS coaching focus group interview guide [file mmc1.docx]

| 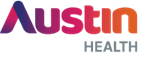 | **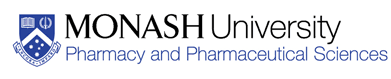** |
| --- | --- |
| **Coaching ward pharmacists to improve antimicrobial prescribing**  **Focus group interview – moderator guide** | |

| **Discussion focus** | **Questions** |
| --- | --- |
| **Welcome** | - Thank you for coming and agreeing to participate - Introduce yourself |
| **Purpose** | - This focus group discussion is designed to gather your experiences, perceptions and opinions about ward pharmacist coaching as a mode of delivering education to improve your knowledge, skills and confidence in AMS. - The focus group discussion will take no more than 60 minutes and will be recorded |
| **Anonymity** | - The recording will be transcribed word for word and then destroyed. - The transcribed notes of the focus group will contain no information that would allow you to be linked to specific statements. - If there are any questions or discussions that you do not wish to answer or participate in, you do not have to do so; however please try to answer and be as involved as possible. |
| **Focus group rules** | - Only one person speaks at a time - There are no right or wrong answers - You don’t have to agree with the views of other people in the group - Does anyone have any questions before we start? |
|  | |
| **Knowledge** | - Has coaching improved your knowledge in antimicrobial use?   - PROBE: Give examples of what new information/knowledge you have learned |
| **Skills** | - Has coaching improved your skills in AMS?   - PROBE: Give examples of what new skills you have learned |
| **Beliefs about capabilities** | - Has coaching improved your confidence in antimicrobial use or making AMS interventions?   - PROBE: Post coaching, which AMS interventions do you feel more confident in identifying and/or discussing with the prescriber?   - PROBE: Post coaching, which areas do you still feel a lack of confidence or were not adequately taught - Has coaching improved your practice in AMS?   - PROBE: Explain how you have changed your practice after the education |
| **Coaching** | - How does coaching compare to other forms of AMS education such as lectures, guidelines, pocket cards etc. that you have received in the past? - What did you think about the frequency and duration of the coaching sessions?   - PROBE – Did you think it was enough, too much or too little? - Did coaching impact on your workload?   - PROBE – did you think the time spent on coaching was manageable or did it adversely impact on your ability to get your work done? - Did you think the coach was appropriate for the role?   - PROBE – did you think the coach had the right expertise in AMS?   - PROBE – how did the coach engage you in learning? Did the coach demonstrate good communication, listening, empathetic and patient skills? - How can this mode of education delivery be improved? |
|  | |
| **Conclusion** | - Are there any other comments that you would like to make? - Thank you for participating. Your opinions will be an asset to the study |
